# Circular RNAs in inflammatory bowel disease

**DOI:** 10.3389/fimmu.2023.1307985

**Published:** 2023-12-22

**Authors:** Jie Lun, Jing Guo, Mengchao Yu, Hongwei Zhang, Jing Fang

**Affiliations:** ^1^ Department of Oncology, The Affiliated Hospital of Qingdao University, Qingdao Cancer Institute, Qingdao, China; ^2^ Central Laboratories, Qingdao Municipal Hospital, Qingdao, China; ^3^ Shandong Provincial Maternal and Child Health Care Hospital Affiliated to Qingdao University, Jinan, China

**Keywords:** circular RNA, inflammatory bowel disease, inflammation, intestinal epithelial barrier, pathogenesis

## Abstract

Inflammatory bowel disease (IBD) is a term encompassing a few chronic inflammatory disorders that leads to damage of the intestinal tract. Although much progress has been made in understanding the pathology of IBD, the precise pathogenesis is not completely understood. Circular RNAs (circRNAs) are single-stranded, covalently closed, endogenous molecules in eukaryotes with a variety of biological functions. CircRNAs have been shown to have regulatory effects in many diseases, such as cancer, cardiovascular disease, and neurological disorders. CircRNAs have also been found to play important roles in IBD, and although they are not sufficiently investigated in the context of IBD, a few circRNAs have been identified as potential biomarkers for the diagnosis and prognosis of IBD and as potential therapeutic targets for IBD. Herein, we survey recent progress in understanding the functions and roles of circRNAs in IBD and discuss their potential clinical applications.

## 1 Introduction

Inflammatory bowel diseases (IBD) are chronic inflammatory disorders that affects the entire gastrointestinal tract. There are two major categories of IBD, i.e., Crohn’s disease (CD) and ulcerative colitis (UC). As the incidence of IBD is increasing over time, it has become a concern around the world (1, 2). IBD was initially thought to predominantly affect people of developed Western countries. In the last few decades, the incidence of IBD is continuously increasing, particularly in recently developed and developing countries (3, 4). The rise of IBD has been in parallel with industrialization and major shifts in the environment and life-style (1). Currently, the antibiotics, anti-inflammatory agents and surgery are employed in the treatment of IBD. The effectiveness of these treatments is variable and usually unsatisfactory (5, 6). By now, IBD cannot be cured and it has a serious impact on the quality of life. Thus, there is an unmet medical need for this disease. The pathogenesis of IBD is not well understood. Much evidence indicates that IBD is probably caused by the combine effect of genetic factors, environmental factors, and the intestine microbiota, which leads to disruption of the intestinal epithelial barrier (7). However, the extent and underlying mechanisms are not well known. Current treatments for IBD mainly focus on treating symptoms with less attention to the repair of mucosal epithelial (8). Conventional therapeutic approaches may not lead to the desired results. Efforts have been made to explore new treatment methods to improve intestinal barrier function (9). Recognizing the pathology of IBD will certainly help develop more efficient treatment methods to ease patient symptoms.

Circular RNAs (circRNAs) are noncoding RNAs with covalently closed loop structures but with no 5′ caps or 3′ poly (A) tails (10). Owing to their circular structure, circRNAs are protected from exonuclease-mediated degradation except under conditions of excess exonuclease levels (11). CircRNAs generally stay in the cytoplasm (12, 13). CircRNAs are abundantly expressed and highly conserved across species (14), and they often exhibit cell type-specific and tissue-specific patterns (15). Thousands of circRNAs have been identified in eukaryotes (16, 17), and some of these circRNAs have been found to play important roles in physiological and pathological processes (18–20). CircRNAs are implicated in various diseases such as chronic inflammatory diseases, metabolic disorders, neuro-disease, cardiovascular diseases, and cancer (21–24). Due to the intrinsic circular characteristics, circRNAs are quite stable both inside cells and in extracellular plasma such as blood and saliva (25–27). CircRNAs can be transported from the cell body to the extracellular environment by exosomes (28) and are expressed in bodily fluids (29). These findings suggest that the disease-associated circRNAs are promising diagnostic biomarkers. In fact, a few circRNAs have been identified as potential diagnostic biomarkers (30). Given that circRNAs are highly stable and specifically expressed, they are also promising and effective targets for multiple diseases treatment (14, 31). A few approaches, such as antisense oligos, RNA interference, small molecules, and the CRISPR/Cas9, have been suggested on basis of the functions of circRNAs (14, 32).

In IBD, circRNAs are not well characterized, and their functions are not well understood. Although circRNAs are not widely investigated in IBD, research results have indicated that these molecules play critical roles in this disease (33). Differential expression of some circRNAs between IBD patients and healthy people has been found (34). Some of these circRNAs exerted regulatory effects on IBD development (33, 35). Increasing evidence shows that circRNAs have potential applications in the diagnosis, prognosis, and therapy of IBD (33, 36). Undoubtedly, more research will be focused on the functions of circRNAs on IBD. Herein, we try to review the current knowledge of the roles of circRNAs on IBD and discuss their potential use in clinical practice.

## 2 Overview of IBD and circRNAs

### 2.1 Basic knowledge of IBD pathogenesis

The pathogenesis of IBD is not well understood. Many studies have shown that IBD is a multifactorial disease caused by a few factors, including genetic defects, environmental abnormalities and dysbiosis, which can disrupt the intestinal homeostasis and trigger immune-mediated inflammation in the gut (7, 37–39).

**Figure 1:**
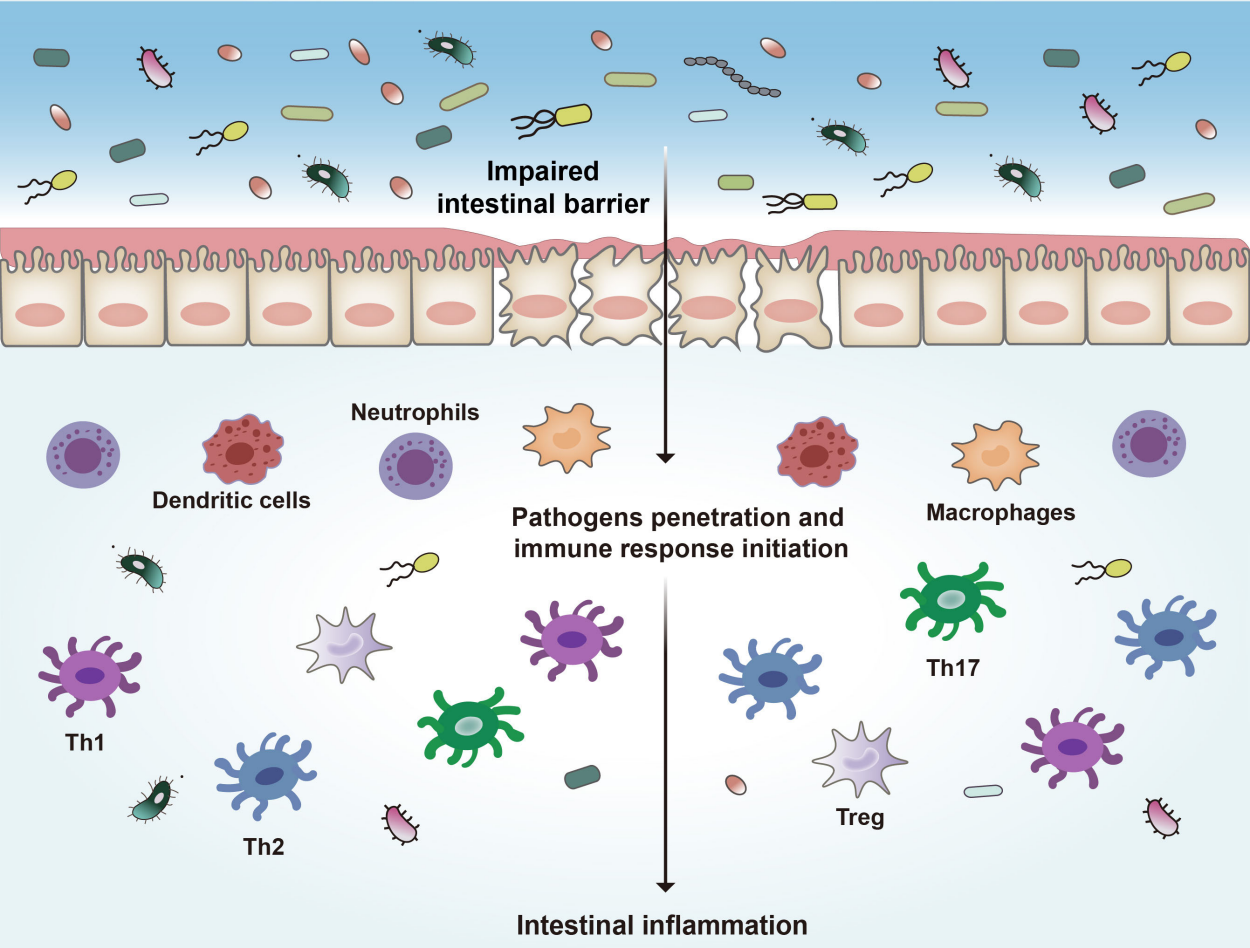
Pathogenesis of IBD. Disruption of the intestinal epithelial barrier leads to the invasion of bacteria and other antigens, which triggers a series of inflammatory reactions and ultimately leads to intestinal inflammation. Th1, type 1 T helper cells; Th2, type 2 T helper cells; Th17, type 17 T helper cells.

One potential mechanism proposed in IBD development is loss of intestinal barrier function, which enables exposure of immune cells to luminal contents and disruption of immune homeostasis. The intestinal barrier consists of intestinal epithelial cells (IECs) and immune cells. IECs include enterocytes (adsorptive cells), goblet cells, neuroendocrine cells, Paneth cells, Microfold cells (M cells), and the tuft cells (40). The most abundant cells are the enterocytes and goblet cells. Goblet cells secrete mucus to cover the epithelium, which is essential to mucosal defense and repair (41). The gut epithelial barrier composes a thick mucosa layer associated with IECs. It was demonstrated that deletion of mucin 2, a major mucin secreted by goblet cells, led to spontaneous colitis in murine models (42). IECs join to each other by tight junction (TJ) that holds epithelial cells together to maintain the integrity of normal intestinal barrier (9, 43). Intestinal TJ proteins help to form a permeable seal which plays a critical role in paracellular transport pathways (44, 45). These key components get together to create a physical barrier to pathogens. The intestinal epithelial barrier separates the gut lumen from the immune system in mucosa, and the impairment of this barrier is thought to be the core mechanism of IBD. Once the epithelial barrier is disrupted, the immune cells will be exposed to microorganisms and other antigens from the lumen (**Figure 1**), which will result in progressive inflammation and damage to the integrity of the intestinal barrier (46–49).

Many immune cells, such as neutrophils, dendritic cells (DCs), macrophages, and lymphoid cells, are in the intestinal epithelium (50, 51). These immune cells complement the physical and functional barrier of IECs and contribute to the immune responses in IBD (39, 52). In response to the disruption of the intestinal barrier, microbial products and other antigens pass through the barrier and encounter DCs and other antigen-presenting cells (APCs), which will initiate proinflammatory and anti-inflammatory signaling pathways and activate different subsets of local and circulating lymphocytes (53, 54). These APCs link innate immunity and adaptive immunity by secreting cytokines and presenting antigens to the T cells (55–57). In response to pathogens the type 1 T helper (Th1) cells are activated to mediate cell-mediated immunity and delayed-type hypersensitivity reactions (58, 59). Th1 cells are induced by IL-12 and secrete IFN-γ, TNF-α, and IL-2, leading to recruitment of macrophages and neutrophils (60). Abnormal Th1 responses are thought to be associated with intestinal inflammation. Th2 cells are induced by IL-4 and secrete IL-4, IL-5, and IL-13, leading to the inhibition of the development of Th1 cells, and enhance the innate immune response through activating macrophages (58). Th17 cells produce IL-17A, IL-17F, IL-21 and IL-22, which are important in driving intestinal inflammation (61, 62). Regulatory T cells (Tregs) are also involved in IBD pathogenesis (63). These cells play a negative role in modulating immune tolerance by producing anti-inflammatory factors including IL-10 and TGF-β (64).

There is cooperation between the immune cells and intestinal epithelial cells in the maintenance of microbiota homeostasis and protection of intestinal barrier. The plasma cells residing in the intestinal lamina propria secrete immunoglobulin A (IgA). The secreted IgA reaches the polymeric immunoglobulin receptor (pIgR) on intestinal epithelium via simple diffusion for transcytosis into the intestinal lumen, where IgA assures the homeostasis of microbiome and protects the host against enteric infections (65–67).

Intestinal barrier homeostasis is reinforced by the resident microbiota, an essential component that maintains the intestinal homeostasis (68, 69). Resident microorganisms influence many physiological processes of the host, including digestion and metabolism, regulation of epithelial barrier and host immune system homeostasis, and inhibition of pathogen colonization (70–73). In the intestine, most of microorganisms are living in a mutualistic relationship with the host, and some symbiotic microorganisms may cause disease in certain conditions (39). The composition of the gut microbiota is believed to be a crucial determinant of host susceptibility to intestine inflammation (39). Change of the gut microbiota composition, i.e., dysbiosis, is a key feature of IBD (74). The altered composition of the intestinal microbiota will break the balance between beneficial and deleterious microbial metabolites in combination with increased intestine permeability and intestine inflammation (73). Dysbiosis of the commensal microorganisms and disruption of the intestinal barrier are believed to be major mechanisms underlying IBD development (39).

There is bidirectional relationship between microbiota and intestinal immune system. The gut microbiota has immunomodulatory effects (75). Gut microbiota abnormality driven innate immunity is a central process in the pathogenesis of IBD (38). The intestinal immune response is an outcome of comprising gut microbiota and immune cell (52). Probiotic intervention maintains a balance between pro- and anti-inflammatory response and affects production of pro- and anti-inflammatory factors in IBD patients. Modulating the microbiota-immune axis may lead to new treatments for IBD. For instance, administration of *Bifidobacterium infantis* reduced the production of pro-inflammatory cytokines such as C-reactive protein, TNF-α, and IL-6 in UC patients (76). It remains unclear that whether circRNAs affect microbiota directly. Considering the influences of circRNAs on immune cells and intestinal epithelial cells, it is possible that circRNAs affect microbiota indirectly, probably through immune system and epithelial cells. And vice versa, the microbiota may also affect circRNAs expression in intestinal epithelial cells and immune cells.

### 2.2 Biogenesis and functions of circRNAs

**Figure 2:**
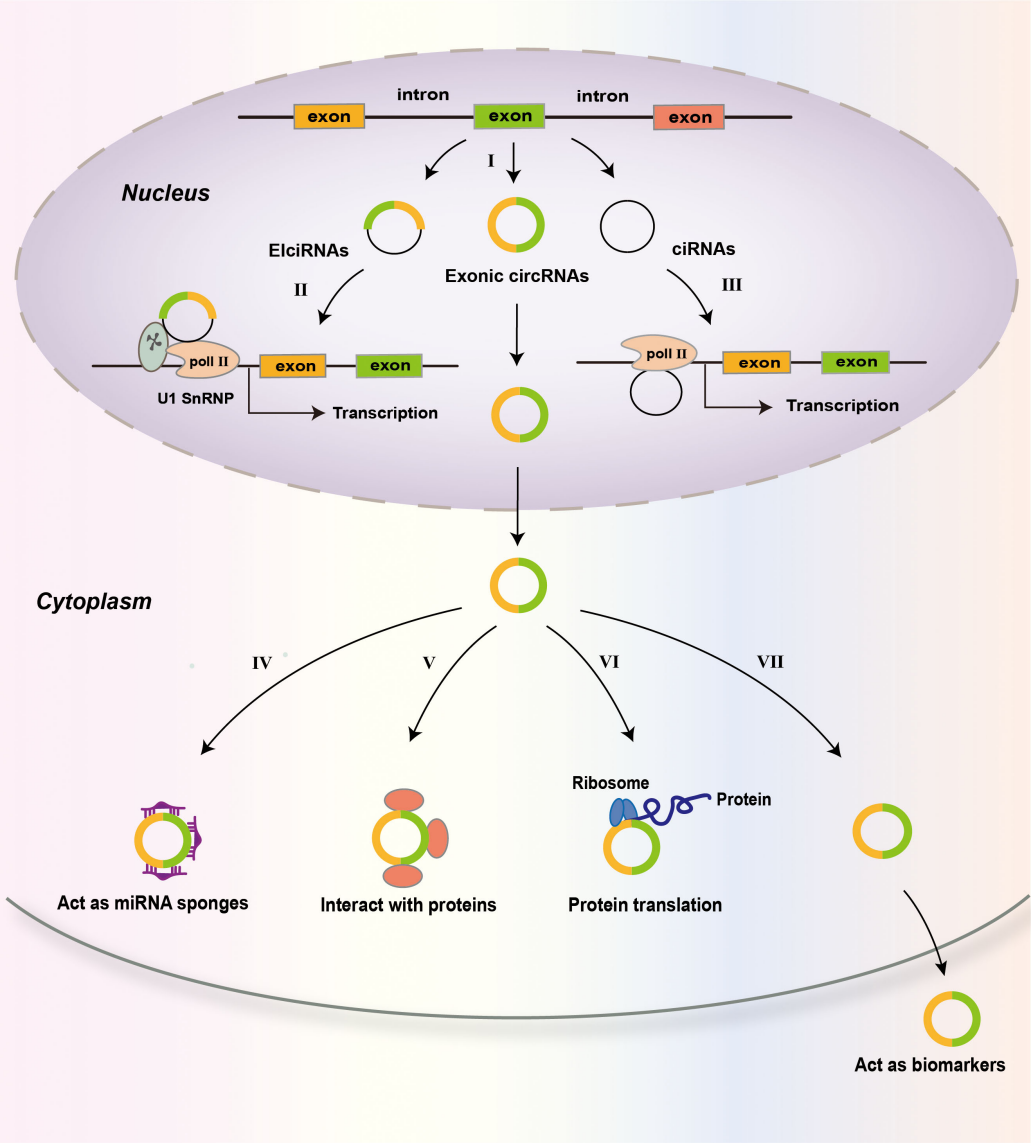
Biogenesis and functions of circRNAs. CircRNAs are predominantly the products of back-splicing events that connect an exon to a preceding exon, resulting in a covalently closed exonic circRNA **(I)**. Retention of internal introns may lead to the production of exon–intron circRNAs (EIcircRNAs) that contain sequences of exons and introns. The intronic lariats that are not branched form circular intronic RNAs (ciRNAs). EIcircRNAs associate with RNA pol II and enhance the transcription of their parental genes via an interaction with U1 snRNP **(II)**. CiRNAs accumulate in the nucleus to regulate gene transcription in cis by promoting Pol II activity on their parental genes **(III)**. In the cytoplasm, circRNAs may act as miRNA sponges **(IV)**; interact with proteins, including RNA-binding proteins (RBPs) **(V)**; and be translated into proteins **(VI)**. CircRNAs may also be used as biomarkers of disease **(VII)**.

CircRNAs are produced from precursor mRNAs (pre-mRNAs) via back-splicing (27, 77). The great majority of circRNAs are encoded by protein-coding genes. CircRNA consists of one or multiple exons (78) (**Figure 2**). For some circRNAs, exons are circularized with introns retained between exons and these circRNAs are termed exon-intron circRNAs or EIciRNAs (79). There is another kind of circRNAs called circular intronic RNAs (ciRNAs). CiRNAs are produced from intronic lariats that escape debranching (80). Exon-only circRNAs are mainly located in the cytoplasm (12, 78), while ciRNAs prefer to localize in the nucleus (80, 81). Compared to their cognate linear RNAs, many circRNAs are expressed at lower levels. However, for some genes, the expression of circRNAs is in high levels (82). A few circRNAs are abundantly expressed in platelet-derived extracellular vesicles (83).

Many studies have demonstrated that some abundant circRNAs act as sponges of microRNA (miRNA) (84). Many circRNAs have one or more miRNA-binding sites, and some circRNAs have sites for multiple miRNAs to bind (21). As miRNA sponges, circRNAs sequester and repress miRNA functions (85). For example, the circular RNA ciRS-7 has more than 70 binding sites for miR-7, and it sponges miR-7 to suppress miR-7 activity, resulting in increased expression of miR-7 target genes (84). Moreover, circRNA adsorption may alter the expression of miRNAs. For instance, ciRS-7 knockout led to decreased levels of mature miR-7 in mice, suggesting that ciRS-7 functions to protect miR-7 from degradation (86). The inhibition or protection of miR-7 by ciRS-7 is cellular context dependent. The expression levels of many circRNAs are very low and many circRNAs have few sites for miRNA binding (78, 87), therefore, these circRNAs may not function as miRNA sponges.

CircRNAs can bind proteins and many circRNA-associated proteins have been identified including RNA-binding proteins (RBPs) (21, 27). CircRNA association with RBPs may regulate the biogenesis, nuclear export, degradation, and function of circRNAs (88, 89). Through interaction, circRNAs may influence the behaviors or impact the fates of associated proteins (89–91). In addition to functioning as protein sponges (11, 92), circRNAs can function as protein scaffolds (93, 94) and protein recruiters (95), thereby regulating protein subcellular localization, protein–protein interaction, stability, and protein-DNA interaction. For instance, in hepatocellular carcinoma (HCC) cells, circRPN2 binds enolase 1 (ENO1) and accelerated ENO1 degradation, thereby regulating cell glycolysis (96). CircAmotl1 enhanced the expression and nuclear translocation of Stat3, which accelerated the healing process in a mouse excisional wound model (97).

Although most circRNAs locate in the cytoplasm, the EIciRNAs and ciRNAs are retained in the nucleus, where they interact with the U1 small nuclear ribonucleoprotein to promote the transcription of their parental genes (79). They can also do so by positively modulating RNA polymerase II (Pol II)-mediated transcription (80) (**Figure 2**). The great majority of circRNAs are believed to be noncoding RNAs. However, recent studies show that some circRNAs can be translated into proteins in a cap-independent way under specific conditions (98, 99).

## 3 Roles of circRNAs in IBD

CircRNAs are implicated in many biological processes and diseases. It was shown that a subset of circRNA quantitative trait loci (circQTLs) SNPs was highly linked to genome-wide association study signals of complex diseases, especially schizophrenia, IBD, and type II diabetes mellitus (100). As regulators of gene expression, circRNAs affect genetic variation and phenotypic changes. Some circular RNAs are found to play roles in the development of IBD (**Table 1**). Although many circRNAs have been found in humans, their roles in IBD are not widely investigated. Analyzing circRNA profiles and investigating their functions in IBD will disclose the mechanisms underlying IBD.

**Table 1:** CircRNAs that are involved in IBD.

Name	Diseases	Tissues/Cells	Expression	Functions and Mechanisms	Clinical Significance	References
CircHECTD1	UC	Colonic tissues	Decrease	Mitigates UC by promoting HuR-dependent autophagy via miR-182-5p	Diagnostic and prognostic biomarker	101
Circ_0001187	UC	Colonic tissues	Increase	Facilitates UC progression via the miR-1236-3p/MYD88 axis	Diagnostic marker and therapeutic target	102
CircHIPK3	CD, UC, sepsis	Gut mucosa	Decrease	Increases the intestinal epithelium repair rate by sponging miR-29b	Therapeutic target	103
CircRNA_103765	CD, UC	PBMCs	Increase	Promotes apoptosis by inhibiting miR-30-mediated DLL4 expression	Therapeutic target	104
CircGMCL1	CD	Colonic tissues	Decrease	Inhibits pyroptosis and proinflammatory cytokines via the miR-124-3p/ANXA7 axis	Therapeutic target	105
Circ_ CCND1	UC	Colonic tissues	Decrease	Promotes Caco-2 cell survival and inhibits inflammatory responses via the miR-142-5p/NCOA3 axis	Therapeutic target	106
CircRNA_102610	CD	PBMCs	Increase	Promotes the EMT by sponging miR-130a-3p	Therapeutic target	107
CircRNA_103516	CD, UC	PBMCs	Increase	Proinflammatory functioning	Diagnostic biomarker	108
CircRNA_102685	CD	Colonic tissues	Increase	Participates in the apoptosis, Toll-like receptor and p53 signaling pathways	Diagnosis and therapy	109
CircCDKN2B-AS1	UC	Colonic tissues	Decrease	Potentially antagonizes colonic epithelial barrier function	Unclear	110
Circ_0007919	UC	Colonic tissues	Decrease	Affects epithelial integrity by regulating hsa-let-7a and hsa-miR-138 target gene expression	Diagnostics and therapeutics	111
CircAtp9b	UC	Plasma	Increase	Promotes apoptosis in colonic epithelial cells by increasing PTEN	Diagnostics and therapeutics	112
Circ_0001021	UC	Colonic tissues	Decrease	Regulates epithelial barrier function by sponging miR-224-5p	Therapeutic target	113
CircRNA_004662	CD	PBMCs	Increase	Unclear	Diagnostic biomarker	114
CircSMAD4	Experimental colitis	Colonic tissues	Increase	Impairs TJ proteins and increases the apoptosis rate by sponging miR-135a-5p to regulate Janus kinase 2 expression	Therapeutic target	115
CircKcnt2	Experimental colitis	ILC3	Increase	Suppresses IL-17 expression	Unclear	116
CircZbtb20	Experimental colitis	ILC3	Increase	Inhibits the m6A modification of Nr4a1 mRNA	Unclear	117

CD, Crohn’s disease; EMT, epithelial mesenchymal transition; ILC3, Group 3 innate lymphoid cells; TJ, tight junction; PBMCs, peripheral blood mononuclear cells; UC, ulcerative colitis.

### 3.1 CircRNAs and intestinal epithelial barrier

The intestinal epithelial barrier is the first line of defense in the body. The intestinal epithelium is composed of a single layer of epithelial cells that tightly link together through junction proteins, and it maintains the integrity of intestinal barrier. The intestinal epithelial barrier protects the host against pathogens in the intestinal lumen, and its disruption is believed to be the core pathology of IBD. Intestinal stem cells (ISCs) are the dividing cells in the intestinal epithelium that can differentiate into IECs and are responsible for the renewal of the epithelial lining (118, 119). Leucine-rich repeat-containing G protein-coupled receptor 5 (Lgr5)-positive ISCs constitute a subgroup of ISCs. Lgr5^+^ ISCs, through self-renewal and differentiation, repair damaged intestinal epithelium and maintain intestinal homeostasis. Disordered Lgr5^+^ ISCs self-renewal and differentiation may lead to intestinal inflammation (120, 121). The circular RNA circPan3 was overexpressed in human and mouse Lgr5^+^ ISCs (122). In ISCs circPan3 bound the IL-13 receptor subunit (IL-13Rα1) mRNA to promote the expression of IL-13Rα1 and increased ISCs self-renewal. Depletion of circPan3 in human Lgr5^+^ ISCs inhibited the renewal of ISCs, leading to the inhibition of epithelium regeneration (122). Likewise, circPan3 bound and stabilized IL-13Rα1 mRNA in mouse ISCs, allowing the expression of IL-13Rα1 expression. These results indicate that circPan3 is critical for the self-renewal of ISCs. A recent report demonstrated that the circular RNA circBtnl1 suppressed ISCs self-renewal via disruption of *Atf4* mRNA stability (123). CircBtnl1 mediated the decay of *Atf4* mRNA to suppress the transcription of Sox9, which negatively modulated the self-renewal of ISCs.

During autophagy, cytoplasmic pathogens in lysosomes are targeted and degraded (124). ATG16L1 plays a critical role in autophagy and intestinal epithelium homeostasis (125). The RNA-binding protein HuR is a posttranscriptional regulator (126, 127). In the intestinal epithelium HuR and the circular RNA circPABPN1 regulate the expression of ATG16L1 (128). High levels of circPABPN1 blocked the binding of HuR to *Atg16l1* mRNA in IECs and suppressed ATG16L1 translation induced by HuR, reducing the ATG16L1 production rate. By controlling the expression of ATG16L1, the HuR-circPABPN1 interaction regulates autophagy. The results suggest that the HuR/circPABPN1/ATG16L1 axis is involved in the development of IBD (128). A recent study revealed that overexpression of circHECTD1 alleviated UC by promoting HuR-dependent autophagy via the action of miR-182-5p (101). The expression of circHECTD1 was decreased in the colonic mucosa of UC patients. Overexpression of circHECTD1 reduced the number of injuries and attenuated inflammation in the colon by promoting autophagy in dextran sulfate sodium salt (DSS)-treated mice.

The level of circ_0001187 was increased in colonic mucosa from UC patients (102). Circ_0001187 acted as a sponge of miR-1236-3p which targeted MYD88. Deletion of circ_0001187 inhibited TNF-α-induced apoptosis, inflammation, and oxidative stress in human normal colorectal mucosa cells, which was blocked by a miR-1236-3p inhibitor. The results indicated that circ_0001187 promoted the development of UC via miR-1236-3p. It was found that the expression of circ_0001187 was upregulated in the serum exosomes of UC patients. These findings suggest that circ_0001187 may serve as a biomarker and/or therapy target in UC.

Genome-wide profile analyses showed that approximately 300 circRNAs, including circHIPK3, were differentially expressed in mice intestinal mucosa after cecal ligation and puncture (103). The expression level of circHIPK3 was found to be decreased in intestinal epithelial cells of IBD patients. Overexpression of circHIPK3 enhanced intestinal epithelium repair after wounding, whereas depletion of circHIPK3 repressed epithelial recovery. In mice silencing circHIPK3 inhibited the growth of IECs, and overexpression of circHIPK3 promoted intestinal epithelium renewal. Mechanistically, circHIPK3 sponged miR-29b, leading to increased expression of Rac1, Cdc42, and cyclin B1 in IECs. These findings indicate that circHIPK3 contributes to the repair of the intestinal epithelium by reducing the level of miR-29b.

Notably, TNF-α promoted the expression of circRNA_103765 in the CD context, which was reversed by treatment with anti-TNF-α mAb (104). *In vitro* TNF-α treatment induced the expression of circRNA_103765, on which cell apoptosis depended. Depletion of circRNA_103765 prevented TNF-α from inducing apoptosis of human IECs. Moreover, circRNA_103765 sponged the miR-30 family members and enhanced the expression of Delta-like ligand 4 (DLL4). These findings show that circRNA_103765 regulates IBD through sponging miR-30 family members to mediate the expression of DLL4. Inhibition of circRNA_103765 might be a strategy to treat IBD.

The expression levels of circGMCL1 in colonic tissues of CD patients were decreased, and this effect was associated with increased CD-associated inflammatory indices (105). CircGMCL1 induced autophagy to inhibit the NLRP3 inflammasome-induced pyroptosis and proinflammatory cytokines. A mechanistic study indicated that circGMCL1 expression was associated with miR-124-3p level and thus facilitated the expression of the miR-124-3p target *Annexin 7*, an autophagy-associated gene, suggesting that circGMCL1 functions via the miR-124-3p/Annexin 7 axis. Moreover, in an experimental colitis model, treatment of the mice with poly-(lactic-co-glycolic acid)-microspheres carrying oe-circGMCL1 significantly reduced the severity of colitis, indicating that circGMCL1 may serve as a target for CD treatment.

CircRNAs conferred protection to the intestinal mucosa of rats with sepsis. Liu et al. (129) investigated the role of circ_0001105 in intestinal epithelial permeability, oxidative damage, and morphological changes in rats with sepsis and found that circ_0001105 protected the intestinal barrier function of the rats by reducing intestinal inflammation, oxidative damage and YAP1 expression, thereby prolonging the survival of the rats with sepsis. Decreased expression of circDMNT3B was conducive to reduced intestinal mucosal permeability because it sponged miR-20b-5p in septic rats (130). Therefore, circDNMT3B silencing increased the permeability of the intestinal mucosa, caused oxidative damage, and increased the levels of IL-6 and IL-10 in intestinal tissue. Depletion of circDNMT3B increased the apoptosis rate of human intestinal epithelial Caco2 cells treated with lipopolysaccharide (LPS). These results indicate that circDMNT3B protected the rat intestinal epithelial barrier.

Expression levels of circRNA CCND1 was decreased in UC patients and LPS-treated Caco2 cells. CircRNA CCND1 inhibited LPS-induced apoptosis and inflammatory responses in the Caco-2 cells and played a protective role in UC via the miR-142-5p/NCOA3 axis (106). The long non-coding RNA CDKN2B-AS1 is expressed in both linear and circular form (110). CDKN2B-AS1 is expressed in colon epithelial cells and its expression level decreased in patients with UC. Inhibition of expression of linear and circular CDKN2B-AS1 increased barrier function.

Upregulated expression of circAtp9b was observed in plasma samples from UC patients (112). LPS treatment increased the expression of circAtp9b in human colonic epithelial cells, which increased the expression of PTEN, eventually promoting apoptosis in colonic epithelial cells. Expression of circ_0001021 was decreased in UC patients, which was related to UC severity (113). Circ_0001021 regulated epithelial barrier function by sponging miR-224-5p. In experimental colitis, circSMAD4 was upregulated in colonic tissues, and the increased circSMAD4 was correlated positively with increased inflammatory factors (115). Overexpression of circSMAD4 disrupted TJ protein function and increased the epithelial cell apoptosis rate by sponging miR-135a-5p. Si-circSMAD4 treatment ameliorated experimental colitis.

### 3.2 CircRNAs and immune homeostasis

CircRNAs are involved in the pathogenesis of immune-related diseases such as autoimmune diseases, cancer, and infectious diseases (131–133). Many studies have indicated that circRNAs play critical roles in immune responses, particularly in cells in myeloid and lymphoid lineages (100, 134).

CircRNAs play a role in the activation of macrophages (135). CircCdr1as was found to be downregulated in proinflammatory macrophages and increased in anti-inflammatory macrophages, and overexpression of circCdr1as induced the expression of anti-inflammatory markers and enhanced the percentage of CD206+ cells in naïve and proinflammatory macrophages (136).

CircRNAs are also involved in the regulation of DCs. Differentially expressed circRNAs were identified in monocyte-derived DCs from the peripheral blood of autoimmune hepatitis patients (31), indicating that circRNAs might influence the functions of DCs and be involved in the pathogenesis of autoimmune disease. The circular RNA circSnx5 modulates DC-driven immunity and tolerance (137). Melatonin inhibited the activation of bone marrow-derived dendritic cells (BMDCs) from colitis model mice, which was reflected by inhibiting the phagocytotic ability of the cells and decreasing their secretion of pro-inflammatory cytokines (138). RNA sequencing analysis indicated that melatonin promoted the transformation of BMDCs into cells with an immune-tolerant phenotype in DSS-induced colitis mice by influencing noncoding RNAs, including circRNA-0520 and circRNA-2243.

In the intestine the group 3 innate lymphoid cells (ILC3s) play important roles in the regulation of immunity, inflammation, and tissue homeostasis (139, 140). Liu et al. (116) found that circKcnt2 was activated in ILC3s under intestinal inflammation conditions. CircKcnt2 recruited the nucleosome remodeling deacetylase (NuRD) complex onto the promoter of *Batf* to suppress the expression of Batf, a critical regulator of adaptive immunity. This in turn suppressed IL-17 expression and thereby ILC3 inactivation to promote the attenuation of innate colitis. A recent study revealed an important role for circRNAs in the regulation of innate lymphoid cell homeostasis. Specifically, circZbtb20 was highly expressed in ILC3 cells, and the deletion of circZbtb20 reduced the number of ILC3 cell and increased the susceptibility of the cells to bacterial infection (117). CircZbtb20 enhanced the interaction between Alkbh5 and Nr4a1 mRNA, which impaired the m6A modification on Nr4a1 mRNA and promoted mRNA stability. Nr4a1 activates Notch2 signaling, which maintains ILC3 cell homeostasis. Through a circRNA-miRNA-mRNA axis, circRNA_004662 cooperated with the mTOR signaling, which is involved in T cells activation and proliferation, proinflammation restriction, and anti-inflammatory promotion responses in monocytes/macrophages (108).

CircRNAs bind and inhibit double-stranded RNA (dsRNA)-dependent protein kinase (PKR), which is related to innate immunity (141). After viral infection, circRNAs are degraded to release PKR which is thus activated and contributes to immune response. Poly(I:C) stimulation or viral infection stimulates global circRNAs degradation mediated by RNase L, which is required for activation of PKR in early innate immune response. In peripheral blood mononuclear cells (PBMCs) from patients with systemic lupus erythematosus, the expression levels of phosphorylated PKR were increased and those of circRNA was decreased.

The innate immunity is inhibited by circRNAs modified with N6-methyladenosine (m6A) (142). Exogenous circRNAs without m6A modification interact with K63-linked ubiquitin chains and the innate immune receptor RIG-I to promote the polymerization and activation of RIG-I, initiating a downstream antiviral signaling and inducing the activation of interferon-regulating factor 3 (IRF3), thereby promoting the expression of genes in an autoimmune pathway.

CircRNAs regulate the production of proinflammatory and anti-inflammatory cytokines (143). Circ_0007456 influenced HCC susceptibility to natural killer (NK) cells by modulating the expression of ICAM-1 (144). CircUHRF1 secreted by HCC cells inhibited the secretion of IFN-γ and TNF-α by NK cells (145). Non-small cell lung cancer (NSCLC) cell-derived exosomal circUSP7 inhibited IFN-γ, TNF-α, granzyme-B and perforin secretion from CD8+ T cells (146). Circ_0005519 was upregulated in CD4+ T cells in asthma patients by inducing the expression of IL-13 and IL-6 in CD4+ T cells (147). CircTRPS1 from bladder cancer cells modulated CD8+ T-cell exhaustion (148).

CircRNA_103516 was markedly increased during flares compared with that in the remission period of both CD and UC, and the increased circRNA_103516 was associated positively with the disease activity (108). CircRNA_103516 was correlated positively with proinflammatory cytokines and negatively with anti-inflammatory cytokines in UC and CD patients, suggesting that circRNA_103516 has a pro-inflammatory function in IBD. The high universality of circRNA_103516 in patients with stricture and penetrating CD, indicates that this circRNA contributes to stricture and penetrating behavior of CD. Song et al. (149) demonstrated that circRIG-I interacted with DEAD-Box Helicase 3 X-Linked to stimulate the MAVS/TRAF5/TBK1 axis, which activated IRF3-mediated type I IFN transcription and aggravated inflammatory damage in the colon. Qiao et al. (109) showed that circRNA-102685 was highly expressed in the colonic tissues of CD patients and potentially regulated miRNA-146 action, alleviating intestinal inflammation through the activation of NF-κB (150) and influenced the functions of immune cells, such as Tregs and DCs (151). Moreover, circRNA-102685 was found to be involved in the chemokine signaling pathway and apoptosis, which was identified in IBD (152, 153).

Increasing evidence suggests that circRNAs are also involved in the regulation of the differentiation of immune cells. CircHIPK3 was involved in Th2 differentiation in allergic rhinitis (154). It was shown that circINPP4B promoted Th17 differentiation by targeting miR-30a in an experimental autoimmune encephalomyelitis model (155). CircRNA000324 and circRNA000324 might be involved in the regulation of differentiation of CD4+ T cell in patients with type 1 diabetes mellitus (156).

### 3.3 CircRNAs as biomarkers for IBD

There is no gold standard for the diagnosis and monitoring of IBD. Clinical manifestations and endoscopy with histopathological examination are two conventional methods for IBD diagnosis (157). Endoscopy combined with biopsy analyses is the most effective way for the diagnosis of IBD. Although effective, this method is costly and invasive and it requires the expertise of clinicians (158). There is an unmet need to develop alternative noninvasive biomarkers for the diagnosis of IBD. CircRNAs are associated with IBD and a few studies have suggested that circRNAs may be noninvasive and inexpensive biomarkers.

Identifying IBD biomarkers is important for diagnosis and prognosis of this disease and the IBD biomarkers may help predict disease behavior and monitor therapy responses. CircRNAs exist in blood cells and plasma (26, 27, 29), extracellular vesicles (28, 83), saliva (25), and urine samples (23). They are stable in tissues and bodily fluids (159). These characteristics indicate that circRNAs are promising biomarkers, and in fact some circRNAs have been identified as potential biomarkers for IBD (**Table 1**). Yin et al. (114) showed that the expression of 4 circular RNAs (004662, 102610, 103124, and 092520) were significantly increased in the PBMCs of CD patients compared with the levels of these circRNAs in healthy controls. The area under the ROC (AUC) values of these four circRNAs were 0.85, 0.78, 0.74, and 0.66, respectively, making them potential diagnostic biomarkers of CD. Furthermore, compared with the level in patients with UC, circRNA_004662 was more highly expressed in patients with CD, suggesting that it may be used to differentiate patients with CD from those with UC.

CircRNA_103516 was upregulated in PBMCs of UC and CD patients and showed a positive correlation with disease activity and the levels of the inflammatory cytokines TNF-α and IFNγ and a negative correlation with the anti-inflammatory cytokine IL-10 level (108). The AUC values of circRNA_103516 used for CD and UC diagnosis were 0.790 and 0.687, respectively. Thus, circRNA_103516 in PBMCs might act as a biomarker for IBD diagnosis. Wang and colleagues (111) recently showed that a reduction in the level of circ_0007919 in patients with UC increased the expression of enhancer of polycomb homolog 1 (EPC1) and vasoactive intestinal polypeptide receptor 1 (VIPR1) because this circRNA sponges hsa-let-7a and miR-138, resulting in intestinal inflammation through SIRT1 inhibition and NF-κB activation. The expression level of circ_0007919 is related to the pathogenesis and progression of UC, indicating that it may have potential application in diagnostics and therapeutics. CircRNA_102610 is a markedly overexpressed circRNA in PBMCs of patients with CD (114), and it promotes the proliferation and epithelial-mesenchymal transition of IECs by absorbing miR-130a-3p (107). CircRNA_102610 was found at a high level in clinical CD samples, suggesting that it may also act as a novel biomarker of IBD.

Differentially expressed circRNAs in saliva are also potential biomarkers. In saliva of oral squamous cell carcinoma (OSCC), hsa_circ_0001874 and hsa_circ_0001971 were found upregulated, indicating the potential of salivary circRNAs as biomarkers for the diagnosis of OSCC (160). It was shown that expression levels of urinary exosomes derived hsa_circ_0036649 was associated with the degree of renal fibrosis, indicating this circRNA might serve as a biomarker for chronic renal fibrosis (161). Whether there are changes of circRNA levels in saliva and urine samples of IBD patients remains unclear. The studies cited in the manuscript only compared IBD patients with healthy controls and the results indicate that expression levels of circRNAs are correlated with the IBD. There are a few types of gut inflammation that are unrelated to IBD, such as infectious colitis and allergic colitis. Whether the expression levels of these circRNAs are also correlated with infectious colitis and allergic colitis remains unknown. Large scale investigation as well as clinical studies are needed to identify circRNAs that have high specificity for the diagnosis of IBD.

## 4 Conclusion

Over the last decades, IBD has become a global health concern (1). Although much efforts have been made, the precise pathogenesis of IBD is not well understood. Emerging evidence has shown that circRNAs are implicated in the pathogenesis of IBD and some of the circRNAs may have potential for clinical applications. CircRNAs can function as miRNAs sponges to regulate genes expression, which regulates intestinal barrier function and immune response. Studies have shown that circRNA_103516 and circRNA_004662 are found to be highly expressed in PBMCs of CD patients and may serve as biomarkers for diagnosis (108, 114). The research of circRNAs in IBD is still in the early stage and the knowledge of circRNAs in IBD is limited. More focus driven investigation in this field is needed. The molecular mechanisms underlying the influence of circRNAs on IBD should be disclosed. Promising therapeutic approaches need to be explored. Inhibition or promotion of circRNA expression may be beneficial in the treatment of IBD. The development of tissue-specific/cell type-specific antagonists or mimics of circRNAs as well as circRNA delivery method could be the most efficient approach. Currently, many studies are still in the laboratory and have not been extended to clinical practice. Animal studies should be conducted and rigorous clinical trials are required to assess the safety and effectiveness of promising treatments.

There are still some unresolved problems and unanswered questions in circRNA. As the sequence of circRNA overlap with its linear mRNA counterpart, the identification and characterization of circRNAs is a challenge. An accurate description of the amount and classification of circRNAs is still lacking. Currently, the methods for identification, characterization, and assessment of circRNAs all depend on the specific junction site (162). The knowledge about circRNA modulation and its effect on circRNA function is limited. How circRNAs are transported into and excrete outside the cells are not well understood. Undoubtedly, addressing these issues will be much helpful to the development of methods for diagnosis, prognosis, and treatment of IBD.

In summary, circRNAs are becoming promising area of IBD research, and the disclosure the role and the underlying mechanism of circRNAs in IBD will undoubtedly help develop efficient therapeutic methods for IBD.

## Author contributions

JL: Writing – original draft, Writing – review & editing. JG: Writing – original draft, Writing – review & editing. MY: Writing – original draft, Writing – review & editing. HZ: Conceptualization, Writing – original draft, Writing – review & editing. JF: Conceptualization, Funding acquisition, Supervision, Writing – original draft, Writing – review & editing.
